# Simultaneous quantification of bioactive components in Chinese herbal spirits by ultra-high performance liquid chromatography coupled to triple-quadrupole mass spectrometry (UHPLC–QQQ–MS/MS)

**DOI:** 10.1186/s13020-021-00435-0

**Published:** 2021-03-12

**Authors:** Yan Hu, Zhe Wang, Fangbo Xia, Wen Yang, Yuan-Cai Liu, Jian-Bo Wan

**Affiliations:** 1 State Key Laboratory of Southwestern Chinese Medicine Resources, Chengdu University of Traditional Chinese Medicine, Chengdu, 611137 China; 2 State Key Laboratory of Quality Research in Chinese Medicine, Institute of Chinese Medical Sciences, University of Macau, Taipa, Macao SAR China; 3 Hubei Provincial Key Lab for Quality and Safety of Traditional Chinese Medicine Health Food, Jing Brand Co.,Ltd., Hubei, China

**Keywords:** Chinese herbal spirit, Bioactive components, UHPLC-QQQ-MS/MS, MRM

## Abstract

**Background:**

The Chinese medicinal wine made from herbal medicines became prevalent among Chinese people. The Chinese herbal spirit is composed of several herbal extracts, and has the certain health functions, such as anti-fatigue and immune regulation. The quality evaluation of Chinese herbal spirit is greatly challenged by the enormous and complex components with great structural diversity and wide range of concentration distribution.

**Methods:**

An ultra-high performance liquid chromatography coupled to triple quadrupole mass spectrometry (UHPLC-QQQ-MS/MS) with multiple reaction monitoring (MRM) method was developed to simultaneously determine forty-three bioactive components in the Chinese herbal spirits produced by year 2014 and 2018.

**Results:**

Quantitative results showed that 11 components, i.e.., puerarin (**5**), purpureaside C (**7**), daidzin (**8**), echinacoside (**9**), acteoside (**15**), epimedin B (**22**), epimedin C (**23**), icariin (**24**), eugenol (**27**), chikusetsusaponin iva (**30**) and Z-ligustilide (**40**), significantly decreased along with the increasing years of storage, while 5 compounds, i.e.., geniposidic acid (**1**), protocatechuic acid (**2**), crustecdysone (**14**), daidzein (**18**) and icariside I (**35**), were basically stable in all samples across the years.

**Concusion:**

The established method allowing to simultaneously determined 43 components with wide structural diversity and trace amounts will facilitate the quality control research of Chinese herbal spirits.

**Supplementary Information:**

The online version contains supplementary material available at 10.1186/s13020-021-00435-0.

## Introduction

Chinese medicinal wine, an alcoholic beverage, is commonly produced by soaking precious Chinese medicinal materials. It became prevalent among Chinese people due to its nourishing and tonic functions. A Chinese herbal spirit from Jing Brand Co., Ltd is one of the most popular medicinal wine in China, which is brewed with the faint-scented Xiaoqu liqueur and several authorized herbal extracts under the guidance of traditional Chinese medicine theory, including *Dioscoreae rhizoma, Curculiginis rhizoma, Angelicae sinensis radix, Cistanches herba, Lycii fructus, Astragali radix, Epimedii folium, Cinnamomi cortex*, and *Caryophylli flos*, etc. [1]. This Chinese herbal spirit has health-care functions of anti-fatigue and immune enhancement [1]. It was shown to strengthen the immune system in the Shen-yang deficient rats, which was associated with the activation of hypothalamic-pituitary-adrenal axis [2]. Furthermore, a clinic study indicated that the Chinese herbal spirit could relieve both physical fatigue and mental fatigue of the patients with the fatigued sub-health status [3].

According to enterprise criterion, total saponins, total flavonoids and icariin are current chemical markers for quality control of this herbal spirit. However, its quality evaluation is greatly challenged by the enormous and complex components with great structural diversity and wide range of concentration distribution. More than 150 ingredients were identified from the herbal spirit, including flavonoids, saponins, alkaloids, phenylethanoids, coumarins, anthraquinones and volatile oil, etc. To date, gas chromatography-mass spectrometry (GC-MS) [1, 4, 5] and liquid chromatography-mass spectrometry (LC-MS) [6], have been established to quantify the volatile flavoring substances and active ingredients in the spirit. However, the very limited quantity of ingredients were quantified. Ultra-high performance liquid chromatography coupled to triple quadrupole mass spectrometry (UHPLC-QQQ-MS) operated in multiple reaction monitoring (MRM) mode is an effective quantification method owing to its well-known high sensitivity and specificity, which could avoid the interference from the background matrix [7–9]. It has been successfully utilized to quantify bioactive components in the complex systems [10–15]. In the current study, therefore, an UHPLC-QQQ-MS method was developed to simultaneously quantify 43 bioactive components in the Chinese herbal spirit samples produced by year 2014 and 2018, and their concentrations in different peoduction years were also compared.

## Materials and methods

### Materials and chemicals

The Chinese herbal spirit samples produced by year 2014 (n = 20) and 2018 (n = 20) were kindly provided by Jing brand Co. Ltd (Hubei, China). The voucher specimens were deposited at room temperature and shielded from light at Institute of Chinese Medical Sciences, University of Macau, Macao. Forty-three reference compounds listed in Table 1 were purchased from Baoji herbest Bio-Tech Co. Ltd (Baoji, China). Their chemical structures were shown in Additional file 1: Figure S1, and the purities of reference standards were over 98 % as confirmed by HPLC-UV. HPLC-grade acetonitrile and methanol were purchased from Merck (Darmstadt, Germany). Formic acid was obtained from Aladdin Industrial Inc. (Shanghai, China). Deionized water was purified using a Millipore Milli-Q purification system (Bedford, MA, USA).

**Table 1:** Mass parameters, calibration curves, LOD, LOQ, precision and recovery of 43 investigated analytes by LC-MS/MS with MRM mode

No	Analyte	RT (min)	Transition	Cone voltage (V)	Collision energy (V)	Calibration curve			LOD (ng/mL)	LOQ (ng/mL)	Precision (RSD, %)		Recovery (%, n = 6)
						Equation	R^2^	Range (μg/mL)			Intra-day	Inter-day	
1	Geniposidic acid	2.05	375.40 → 195.24	15	+ 15	y = 260.2x − 43.8	0.9985	0.064–16.46	8.04	32.1	4.44	5.74	94.2
2	Protocatechuic acid	2.26	155.20 → 65.13	30	+ 20	y = 1781.6x + 43.5	0.9995	0.009–9.340	0.65	2.28	2.59	5.15	97.8
3	Chlorogenic acid	3.35	355.30 → 163.08	20	+ 15	y = 19714x − 689	0.9973	0.007–3.575	0.10	0.33	4.89	5.80	90.8
4	Scopolin	3.47	355.11 → 193.05	25	+ 15	y = 20562x − 1022	0.9987	0.005–5.550	0.18	0.60	2.91	3.04	104.2
5	Puerarin	4.03	417.30 → 297.20	46	+ 25	y = 10069x + 1933	0.9954	0.007–13.89	0.03	0.11	2.60	3.13	101.2
6	Magnoflorine	4.45	342.30 → 297.30	42	+ 19	y = 27481x + 1247	0.9992	0.001–5.542	0.25	0.74	1.87	3.99	104.8
7	Purpureaside C	4.64	787.46 → 163.03	20	+ 30	y = 444.3x + 34.7	0.9992	0.022–11.15	5.44	21.7	1.06	3.70	103.1
8	Daidzin	4.76	417.36 → 255.22	30	+ 20	y = 17278x + 4508	0.9991	0.009–36.47	0.04	0.14	1.18	3.38	98.0
9	Echinacoside	4.91	787.55 → 325.10	20	+ 20	y = 317.6x + 5.9	0.9995	0.046–11.78	2.16	7.66	3.38	3.51	90.9
10	Rutinum	5.63	611.20 → 303.10	35	+ 15	y = 12785x + 160	0.9991	0.003–2.600	0.05	0.15	3.17	5.20	95.6
11	Calycosin-7-glucoside	5.70	447.28 → 285.19	36	+ 17	y = 47510x + 2889	0.9962	0.001–5.725	0.02	0.05	2.08	2.41	107.6
12	Ferulic acid	5.74	195.10 → 117.00	20	+ 23	y = 10691x + 201	0.9992	0.004–4.250	0.06	0.24	4.42	5.16	100.6
13	Hyperoside	5.79	465.27 → 303.16	26	+ 15	y = 14954x − 199	0.9994	0.007–3.650	0.09	0.34	2.82	2.85	97.1
14	Crustecdysone	6.04	481.41 → 371.34	30	+ 15	y = 881.7x + 54.6	0.9999	0.017–16.91	4.13	16.5	2.44	3.07	99.7
15	Acteoside	6.07	625.39 → 163.12	20	+ 30	y = 693.3x + 52.6	0.9989	0.015–7.721	3.77	11.3	4.50	5.72	99.4
16	Coumarin	7.41	147.25 → 91.16	30	+ 30	y = 20078x + 89	0.9999	0.001–4.853	0.04	0.19	3.16	3.65	103.4
17	Ononin	8.10	431.26 → 269.26	30	+ 25	y = 31798x + 1102	0.9997	0.002–7.794	0.03	0.10	1.47	3.36	103.9
18	Daidzein	8.19	255.23 → 199.41	30	+ 25	y = 12439x + 90	0.9998	0.001–1.131	0.07	0.28	0.68	3.05	96.3
19	Salvianolic acid A	8.51	495.17 → 223.19	20	+ 40	y = 984.7x − 53.3	0.9980	0.017–8.906	4.35	11.6	0.72	1.79	106.9
20	Quercetin	8.92	303.60 → 229.05	50	+ 30	y = 3438x + 205	0.9990	0.013–13.00	0.71	2.39	3.02	3.45	91.9
21	Epimedin A1	8.98	839.58 → 369.27	36	+ 34	y = 9091x + 223	0.9996	0.007–6.700	0.68	2.18	4.40	4.73	104.5
22	Epimedin B	9.31	809.57 → 369.22	30	+ 34	y = 7352x + 1340	0.9924	0.007–13.80	0.19	0.75	1.08	3.85	99.4
23	Epimedin C	9.48	823.58 → 369.27	34	+ 35	y = 8289x + 32	0.9998	0.005–10.25	0.71	2.11	1.48	1.90	94.8
24	Icariin	9.66	677.48 → 369.22	38	+ 27	y = 12010x + 1110	0.9987	0.002–12.44	0.01	0.03	3.23	4.01	92.2
25	Formononetin	11.88	269.19 → 253.11	40	+ 30	y = 10680x + 39	0.9997	0.001–1.011	0.02	0.06	1.82	2.44	111.7
26	Baohuoside II	12.03	501.29 → 355.24	30	+ 15	y = 20952x − 126	0.9997	0.002–3.456	0.03	0.11	1.47	5.18	106.5
27	Eugenol	12.13	164.07 → 103.13	44	+ 21	y = 185.8x + 134.1	0.9986	0.035–35.52	8.54	27.7	3.63	3.72	90.3
28	Astragaloside A	12.37	785.50 → 455.40	25	+ 15	y = 947.3x + 6.2	0.9996	0.012–3.028	0.80	2.00	3.15	4.29	99.3
29	Rhamnocitrin	12.39	301.10 → 258.10	50	+ 30	y = 17437x − 344	0.9989	0.003–2.900	0.06	0.22	4.54	5.38	106.4
30	Chikusetsusaponin iva	12.74	793.57 → 793.57	30	-20	y = 1110x + 21	1.0000	0.010–9.779	0.30	1.19	3.60	4.10	92.3
31	Geniposide	13.01	389.43 → 255.29	30	+ 25	y = 72.6x + 4.7	0.9998	0.092–11.84	23.1	46.2	3.64	5.15	102.0
32	Sagittatoside A	13.16	677.33 → 369.24	30	+ 15	y = 6603x + 21	0.9999	0.003–2.978	0.73	2.91	2.53	4.49	95.8
33	Astragaloside II	13.31	827.75 → 175.09	20	+ 30	y = 579.6x + 9.0	0.9992	0.004–2.243	0.73	2.19	3.49	5.81	106.2
34	Sagittatoside B	13.45	647.45 → 369.22	24	+ 24	y = 9750x + 95	0.9998	0.004–3.600	0.59	2.34	4.79	4.88	109.1
35	Icariside I	13.48	531.19 → 369.14	35	+ 30	y = 26745x + 119	0.9991	0.000–0.563	0.03	0.07	2.70	3.96	97.5
36	2′'-O-rhamnosyl Icariside II	13.51	661.47 → 369.28	28	+ 21	y = 14713x + 522	0.9979	0.003–5.150	0.11	0.38	3.47	4.15	90.6
37	Baohuoside I	13.83	515.30 → 369.20	26	+ 20	y = 48629x + 525	0.9988	0.000–3.375	0.03	0.10	0.99	2.69	96.8
38	Desmethyl Icaritin	13.87	355.10 → 299.10	50	+ 20	y = 129383x + 2666	0.9991	0.001–3.950	0.03	0.08	2.37	3.43	95.4
39	Astragaloside I	14.11	869.74 → 217.27	15	+ 15	y = 854.4x + 32.2	0.9994	0.009–8.750	1.07	4.27	1.02	1.99	101.4
40	Z-ligustilide	14.73	191.11 → 117.01	38	+ 22	y = 8945x − 92	0.9998	0.005–11.00	0.86	2.94	4.27	4.68	98.8
41	Icaritin	15.38	369.14 → 313.07	50	+ 26	y = 220062x + 3559	0.9959	0.000–0.322	0.00	0.01	3.13	4.85	106.9
42	Tanshinone IIA	16.04	295.29 → 277.24	50	+ 19	y = 64914x + 4212	0.9911	0.001–3.200	0.05	0.17	2.06	2.68	104.9
43	Oleanolic acid	16.69	439.36 → 203.18	35	+ 25	y = 19247x + 370	0.9981	0.002–1.611	0.21	0.53	3.08	3.60	92.3

### Sample preparation and standard solution preparation

An aliquot of 1mL of the spirit was diluted with the equivalent volume of acetonitrile, and vortexed for 1 min. The mixture was centrifuged at 14,800 rpm for 20 min. After the centrifugation, the supernatant was filtered through a 0.22 μm filter (PVDF Millex-GV, 13 mm, Millipore) prior to the quantitative analysis. All of 43 reference standards were accurately weighed and dissolved in methanol to prepare individual stock solutions at the concentrations ranging from 0.58 to 2.29 mg mL^− 1^. The mixed standard solution was prepared by mixing appropriate volumes of the individual stock solutions and further diluted to a series of proper concentrations with methanol.

### LC-MS/MS analysis

Forty bathes of the spirit samples were analyzed by an ACQUITY™ UPLC system (Waters, Milford, MA, US) coupled with a Xevo TQD triple-quadrupole tandem mass spectrometry (QQQ-MS/MS, Waters Co., Manchester, UK). Chromatographic separation was implemented on an ACQUITY UPLC BEH C_18_ column (150 × 2.1 mm, 1.7 μm). The mobile phases were consisted of 0.1 % (*v/v*) aqueous formic acid solution (phase A) and acetonitrile containing 0.1 % formic acid (phase B) at the flow-rate of 0.3 mL min^−1^ using a gradient elution program as follows: 5–40 % B at 0–12 min, 40–100 % B at 12–16 min, isocratic 100 % B for 2 min, and the re-equilibrated by 5 % B for 3 min. The sample injection volume was set at 2 µL. The temperatures of column and injector were set at 35 ℃ and 8 ℃, respectively.

Data acquisition was performed by a Xevo TQD QQQ-MS equipped with an electrospray ionization (ESI) using MRM mode. MS was operated in either the positive (ESI+) or negative mode (ESI-) to obtain satisfactory MS response for 43 investigated compounds due to their different properties. The MS and MS/MS spectra of each compound were acquired using the mixed standard solution. The optimized MS parameters were as follows: capillary voltage, 3.5 kV (positive ion mode) and − 3.0 kV (negative ion mode); source temperature, 140 ℃; desolvation gas flow and temperature, 650 L h^−1^ and 350 ℃; cone flow, 50 L h^−1^. The ion transitions, cone voltage, and collision energy for each compound were optimized and shown in Table 1. All instrumentations were synchronized and controlled by Waters Masslynx software (version, 4.1).

### Method validation

To evaluate sensitivity and precision of the established UHPLC-QQQ-MS/MS method, the linearity, limit of detection (LOD), limit of quantitation (LOQ), precision and recovery of 43 analytes were tested. The calibration curve of each compound was constructed by plotting the peak areas against the concentrations using the mixed standard solution at a series of concentrations. The precision was examined by calculating intra- and inter-day variations of each analyte using the mixed standards for five replicates within a day and three consecutive days. The LOD and LOQ for each analyte were estimated at signal-to-noise ratio (S/N) of about 3 and 10, respectively. The accuracy of the established method was assessed by spike recovery experiments. A known amount (equal to the content for each analyte in the sample) of the mixed standards was spiked into the random spirit sample (S-2018-08). The sample was prepared with six replicates and analyzed by the method mentioned above. The recovery (%) was calculated as the following equation:

$${\text{Recovery }}\left( \% \right)\; = \;{1}00\; \times \;\left( {{\text{detected amount}}\; - \;{\text{original amount}}} \right)/{\text{ spiked amount}}$$

### Statistical analysis

The concentrations of 43 analytes in the spirit samples were presented as mean ± standard deviation (SD). The difference between groups was assessed by student *t*-test using a GraphPad Prism package (version 6.0, San Diego, CA, USA), and a *p*-value of less than 0.05 was considered statistically significant. Orthogonal partial least squares-discriminant analysis (OPLS-DA), a supervised multiple regression, was conducted to discriminate the spirit samples manufactured in different years according to the levels of investigated analytes by SIMCA-P software (version 14.1, Umetrics, Umeå, Sweden).

## Results and discussion

### LC-MS/MS method development

The quality control research related to this Chinese herbal spirit mainly focused on the determination of volatile components by GC-MS [1, 4, 5], and very limited number of nonvolatile bioactive components were quantified by LC-MS [6]. Due to consisting of multiple herbal extracts and the faint-scented Xiaoqu liqueur, the herbal spirit is a very complex matrix containing the numerous organic and inorganic compounds with wide range of concentrations. Thus, the simultaneous quantitation of forty-three compounds with various chemical types encounters the great challenge in short running time using UHPLC. MRM is a highly specific technique for quantifying the targeted analyte, regardless of baseline chromatographic separation. The targeted analyte in chromatographic co-elution could be accurately quantified if they have different MS or MS/MS characteristics. However, the co-eluted analytes may cause the potential mutual ionization suppression in ESI, leading to the low MS response. Therefore, it is also necessary to optimize the chromatographic conditions, including column and mobile phase, to achieve the high sensitivity and fast separation in LC-MS/MS analysis. Three UHPLC columns, such as BEH C_18_ column, BEH HILIC column and HSS T3 C_18_ column, were examined. As results, an ACQUITY BEH C_18_ column was most suitable for the separation of the targeted compounds in the sample owing to the best resolution and the most peak capacity. Furthermore, several types of mobile phases, including methanol/water and acetonitrile/water system supplemented with various modifiers, were tested. The results shown that 0.1 % aqueous formic acid solution / acetonitrile with 0.1 % formic acid was the optimum mobile phases to obtain the chromatogram with the best resolution.

**Fig. 1:**
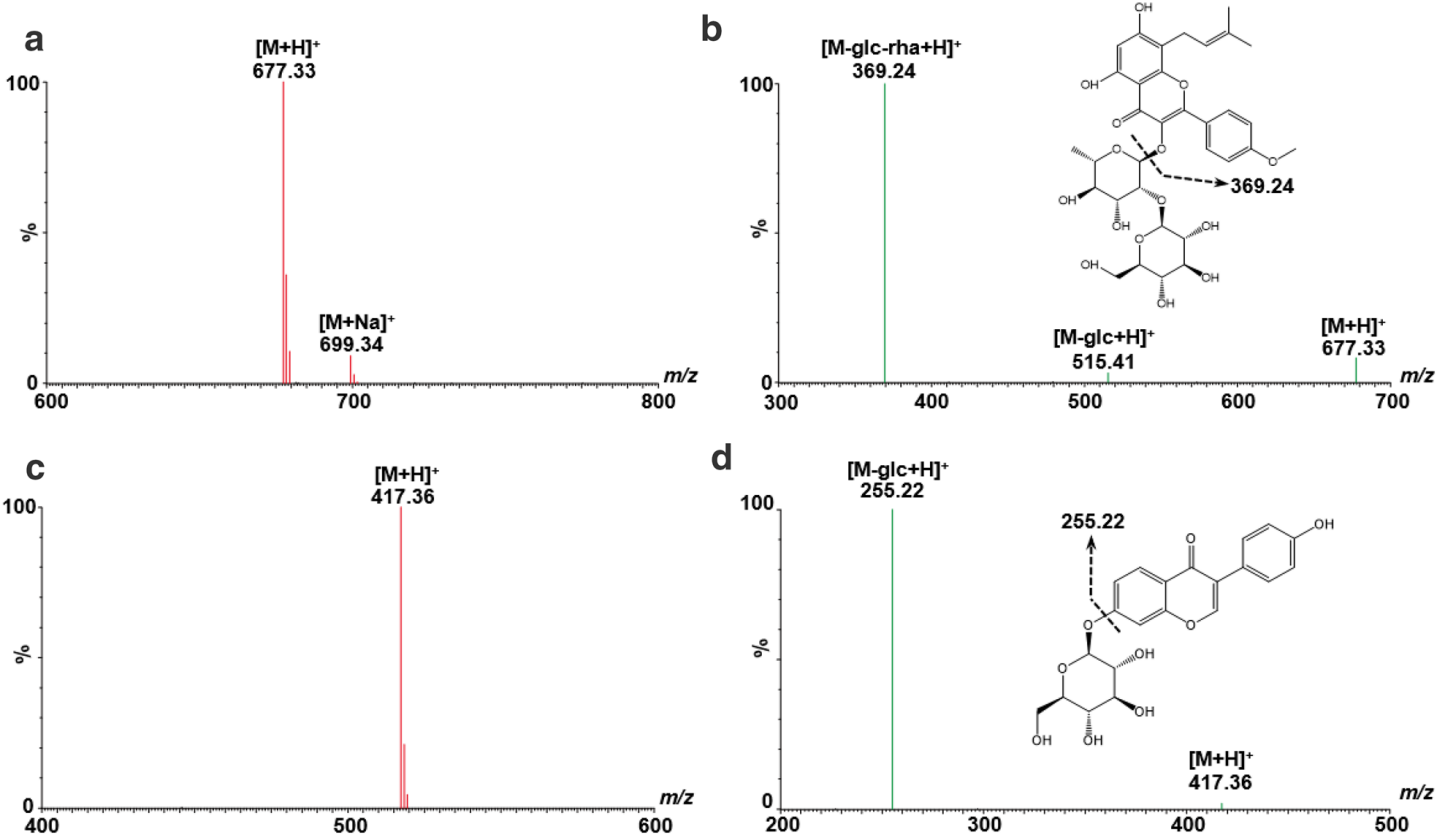
Full scan spectra (left), the corresponding daughter scan spectra and the proposed fragmentation pattern (right) of sagittatoside A (**32**, **a**) and daidzin (**18**, **b**)

**Fig. 2:**
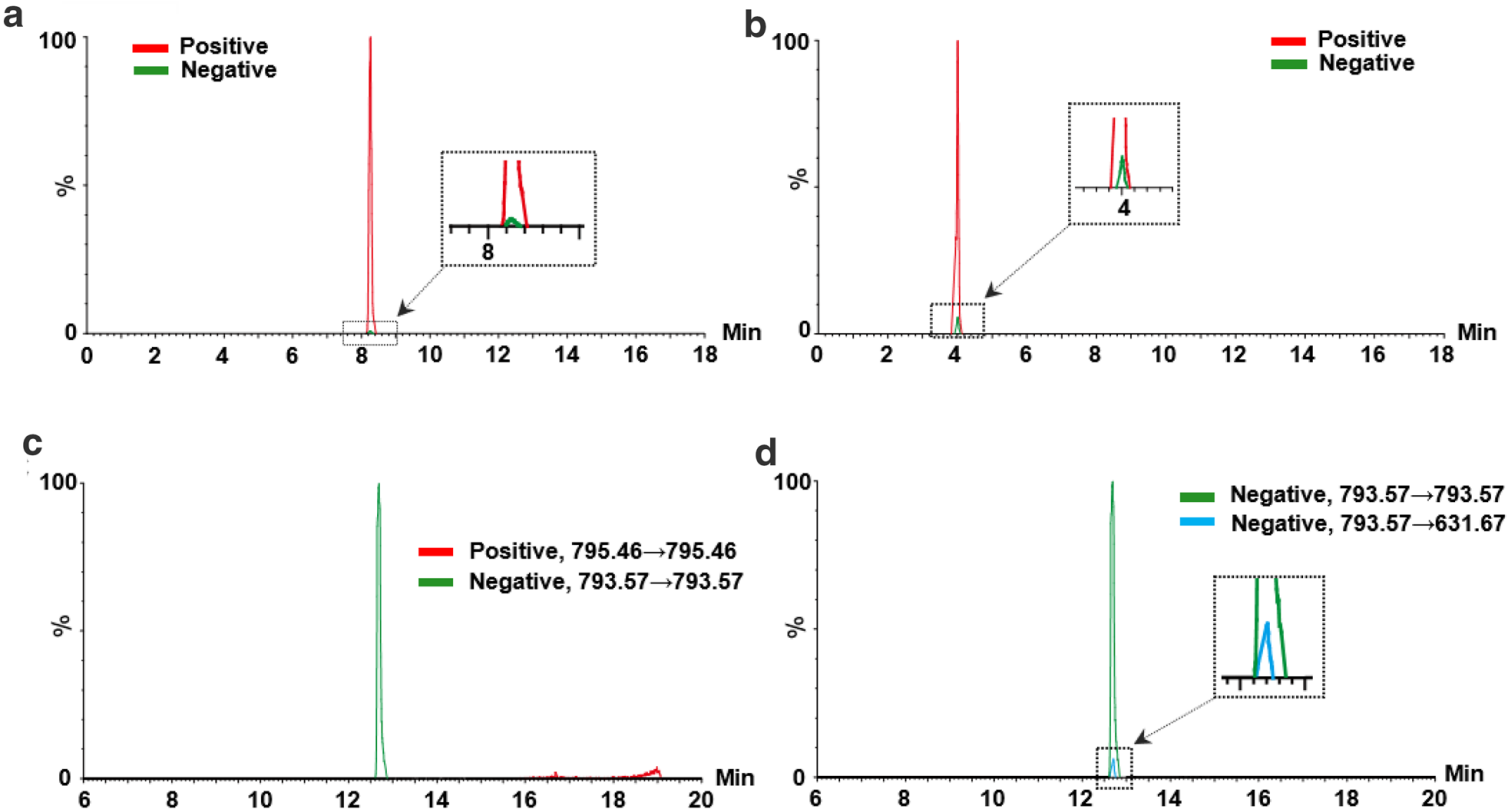
The MRM chromatograms of daidzein (**18**, **a**), puerarin (**5**, **b**) and Chikusetsu saponin iva (**30**, **c**) in both positive and negative ion modes; The MRM chromatograms of Chikusetsu saponin iva (**d**) with different transitions (*m/z* 793.57→793.57 and *m/z* 793.57→631.67) in the negative ion mode

It is critical to design ion transition of each analyte, including precursor ions and their corresponding product ions, in MRM analysis. The full scan was used to select the precursor ions using their reference standards, and the dominated fragment ion in daughter scan was chosen as the corresponding product ion (Fig. 1). Taking sagittatoside A (**32**, MW = 676.24), a main component derived from *Epimedii folium*, as an example, the protonated ion [M + H]^+^ *m/z* 677.33 was presented with the highest abundance in full scan spectrum, the adduct ion [M + Na]^+^ (*m/z* 699.34), was also observed with less intensity. While, the product ion *m/z* 369.24 was dominated in the corresponding daughter scan of [M + H]^+^ (Fig. 1 a, b). The mass difference between parent and product ions was 308.09, which corresponded to the loss of one glucose and one rhamnose units [15]. Herein, the ion transition (*m/z* 677.33 → 369.24) was selected to quantify sagittatoside A in the liqueur by MRM. Likewise, the ion transition (*m/z* 417.36 → 255.22) was optimized to determine daidzin (**8**, Fig. 1 c, d). To achieve maximum signal, both positive and negative ion modes were tested and compared. The results indicated that all compounds, except Chikusetsusaponin iva (**30**), shown the higher sensitivities in the positive ion mode than in the negative mode. For example, MRM chromatograms of daidzein (**18**) and puerarin (**5**) in positive ion mode were remarkably higher than that in negative mode (Fig. 2a, b). However, the higher MS intensity of chikusetsusaponin iva was observed in negative mode (Fig. 2c). Furthermore, the transition of chikusetsusaponin iva in the negative mode were further optimized. We found that multiple ion monitoring (MIM), *i.e. m/z* 793.57 → 793.57, was more suitable for the detection of chikusetsusaponin iva, compared to other transitions, such as *m/z* 793.57 → 631.67 (Fig. 2d).

**Fig. 3:**
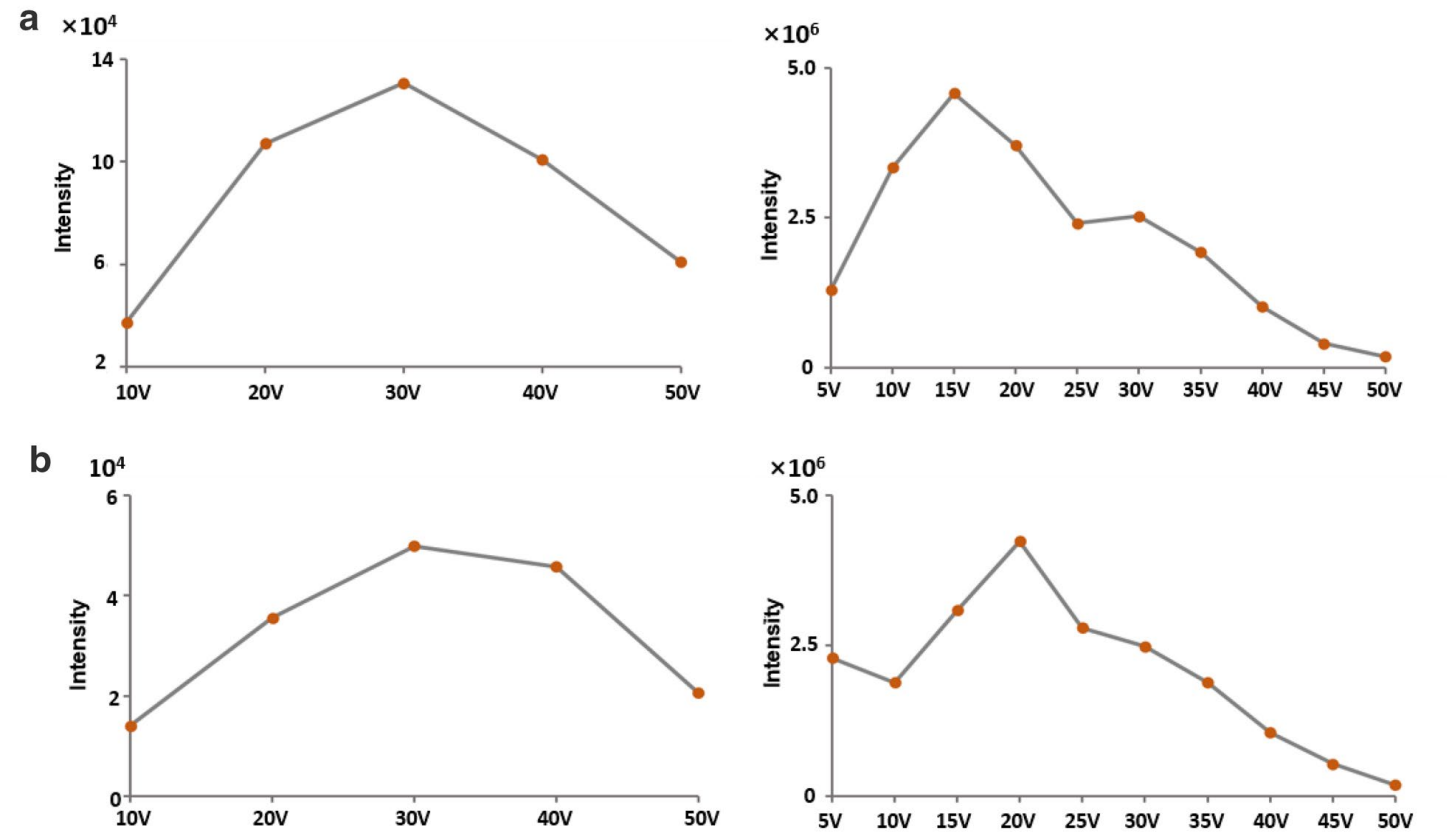
The intensity distributions of the fragment ions derived from sagittatoside A (*m/z* 677.33→369.24, **a**) and daidzin (*m/z* 417.36→255.22, **b**) with the cone voltage ranging from 10 V to 50 V (left), and collision energy ranging from 5 to 50 V (right) in positive ion mode

**Fig. 4:**
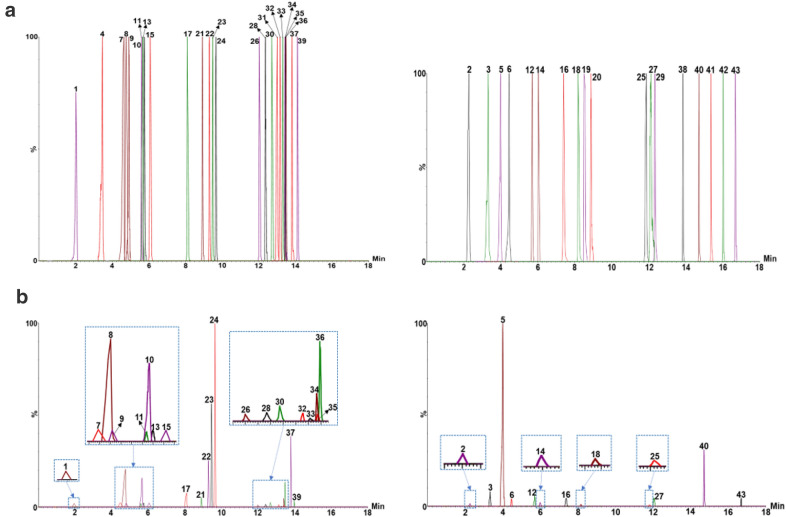
The MRM chromatograms of glycosides (left) and the remaining analytes (right) in the mixed standards (**a**) and the Chinese herbal spirit sample (S-2018-08, **b**)

Additionally, cone voltage (CV) and collision energy (CE), the important factors that affect the sensitivity of UHPLC-QQQ-MS/MS analysis, were also optimized for each analyte using reference standard. CV and CE were optimized from 10 V to 50 V with a step of 10 V and from 5 V to 50 V with a step of 5 V, respectively. Taking sagittatoside A as an example, the signal intensity of product ion m/z 369.24 increased along with CV from 10 V to 30 V or CE from 5 V to 15 V, then decreased with the increasing voltages (Fig. 3a). Therefore, 30 V of CV and 15 V of CE were chosen for the quantification of sagittatoside A. Likewise, 30 V of CV and 20 V of CE were used to determine the daidzin (Fig. 3b). In a similar manner, the mass spectrometry conditions of all analytes were optimized and listed in Table 1. Under the optimized LC-MS/MS conditions, 43 analytes were well separated and detected in 18 min. The representative MRM chromatograms of the mixed standards and the Chinese herbal spirit are shown in Fig. 4.

### Method validation

The established LC-MS/MS method was validated by a series of experiments, including linearity, sensitivity, precision, and accuracy. As shown in Table 1, the calibration curves of all analytes exhibited good linear regression (R^2^ ≥ 0.9911) within the wide dynamic range. The LODs and LOQs of analytes were less than 8.54 and 27.7 ng mL^− 1^, respectively. Other than that of geniposide was 23.1 and 46.2 ng mL^− 1^, respectively, which was higher than other compounds. The overall intra-day and inter-day variations were lower than 4.89 % and 5.81 %, respectively. Additionally, the developed method had the acceptable accuracy with recoveries ranging from 90.3 to 111.7 %. Taken together, the proposed LC-MS/MS method is sensitive, precise and accurate for the simultaneous determination of these 43 compounds in the Chinese herbal spirit produced in different years.

### Quantification of 43 compounds in the Chinese herbal spirit

 The validated method was successfully applied to quantify 43 compounds in the liqueur samples. As shown in Table 2 and 35 of 43 analytes were detected and quantified in the liqueur samples produced in both 2014 and 2018. Eight analytes, including scopolin (**4**), salvianolic acid A (**19**), quercetin (**20**), rhamnocitrin (**29**), geniposide (**31**), desmethyl icaritin (**38**), icaritin (**41**), tanshinone IIA (**42**), were not detected in all samples. Eight compounds, including eugenol (**27**), puerarin (**5**), icariin (**24**), epimedin C (**23**), epimedin B (**22**), chikusetsusaponin iva (**30**), daidzin (**8**) and crustecdysone (**14**) were identified as major components in the liqueur with contents more than 1 µg mL^− 1^. Among them, eugenol (**27**) is a major components with the highest abundance in the liqueur samples. While, daidzein (**18**), formononetin (**25**), baohuoside II (**26**) and icariside I (**35**) were trace components with the content of less than 0.1 µg mL^− 1^. Additionally, 26 analytes were observed to be significantly different between the Chinese herbal spirits produced in year 2014 and 2018 (*p* < 0.05).

**Table 2:** The contents of 43 investigated components in the Chinese herbal spirits (ND = not detected)

No	Analyte	Content (μg/mL)	
		2018	2014
1	Geniposidic acid	1.15 ± 0.22	1.15 ± 0.16
2	Protocatechuic acid	0.37 ± 0.11	0.39 ± 0.09
3	Chlorogenic acid	0.48 ± 0.13	0.28 ± 0.07***
4	Scopolin	ND	ND
5	Puerarin	6.84 ± 2.41	5.44 ± 1.05*
6	Magnoflorine	0.31 ± 0.22	0.11 ± 0.13**
7	Purpureaside C	1.05 ± 1.26	0.08 ± 0.26**
8	Daidzin	2.56 ± 1.12	1.95 ± 0.49
9	Echinacoside	2.20 ± 2.67	0.18 ± 0.54**
10	Rutinum	0.16 ± 0.07	0.07 ± 0.04***
11	Calycosin-7-glucoside	0.42 ± 0.08	0.34 ± 0.06**
12	Ferulic acid	0.31 ± 0.09	0.19 ± 0.04***
13	Hyperoside	0.22 ± 0.08	0.08 ± 0.02***
14	Crustecdysone	2.18 ± 0.33	2.25 ± 0.38
15	Acteoside	0.83 ± 1.19	0.04 ± 0.13*
16	Coumarin	0.26 ± 0.13	0.22 ± 0.06
17	Ononin	0.25 ± 0.12	0.19 ± 0.03*
18	Daidzein	0.06 ± 0.04	0.06 ± 0.04
19	Salvianolic acid A	ND	ND
20	Quercetin	ND	ND
21	Epimedin A1	0.45 ± 0.15	0.27 ± 0.19**
22	Epimedin B	2.89 ± 0.61	1.65 ± 0.51***
23	Epimedin C	6.00 ± 0.92	4.55 ± 1.52***
24	Icariin	7.10 ± 0.91	4.80 ± 0.57***
25	Formononetin	0.00 ± 0.00	0.01 ± 0.00***
26	Baohuoside II	0.10 ± 0.04	0.06 ± 0.02**
27	Eugenol	18.60 ± 4.02	15.53 ± 1.55**
28	Astragaloside A	0.07 ± 0.04	0.13 ± 0.05***
29	Rhamnocitrin	ND	ND
30	Chikusetsusaponin iva	4.15 ± 2.81	2.76 ± 0.35
31	Geniposide	ND	ND
32	Sagittatoside A	0.50 ± 0.22	0.17 ± 0.1***
33	Astragaloside II	0.27 ± 0.17	0.32 ± 0.19
34	Sagittatoside B	0.30 ± 0.12	0.09 ± 0.04***
35	Icariside I	0.02 ± 0.01	0.02 ± 0.01
36	2′'-O-rhamnosyl icariside II	0.39 ± 0.14	0.20 ± 0.44***
37	Baohuoside I	0.35 ± 0.15	0.17 ± 0.09***
38	Desmethyl Icaritin	ND	ND
39	Astragaloside I	0.09 ± 0.07	0.04 ± 0.03*
40	Z-ligustilide	1.47 ± 0.73	0.57 ± 0.16***
41	Icaritin	ND	ND
42	Tanshinone IIA	ND	ND
43	Oleanolic acid	0.14 ± 0.07	0.09 ± 0.03**

*p < 0.05, **p < 0.01 , ***p < 0.001

**Fig. 5:**
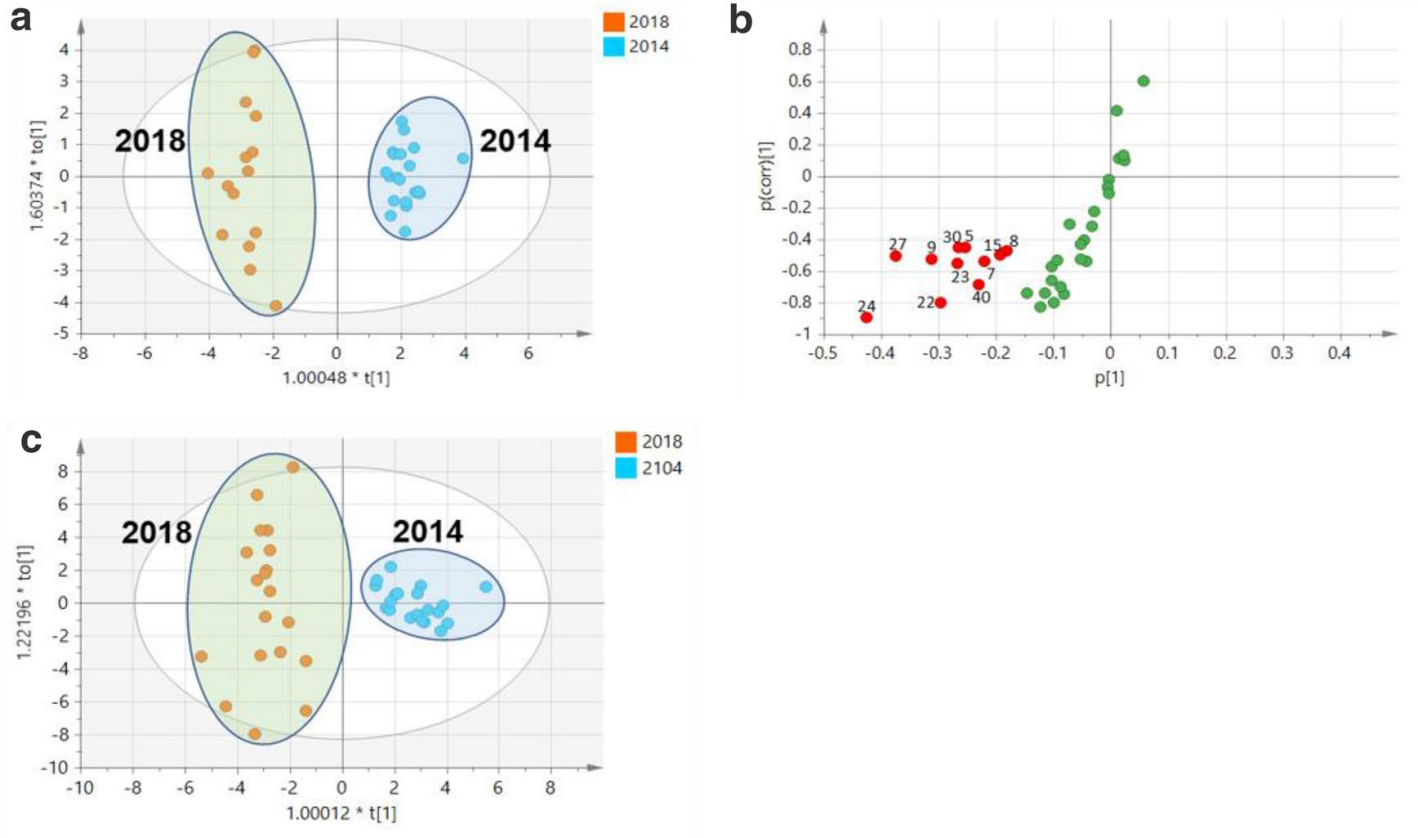
OPLS-DA score plots (**a**) and the corresponding *S*-plot (**b**) based on the contents of 43 analytes investigated in the Chinese herbal spirit samples produced in year 2014 and 2018. OPLS-DA score plot (**c**) according to the contents of 11 highlighted differentiated ions across the samples. The differentiated ions (VIP > 1 and p < 0.05) were marked in red. The compound number in the S-plot represent in the same manner as in Table 1

**Fig. 6:**
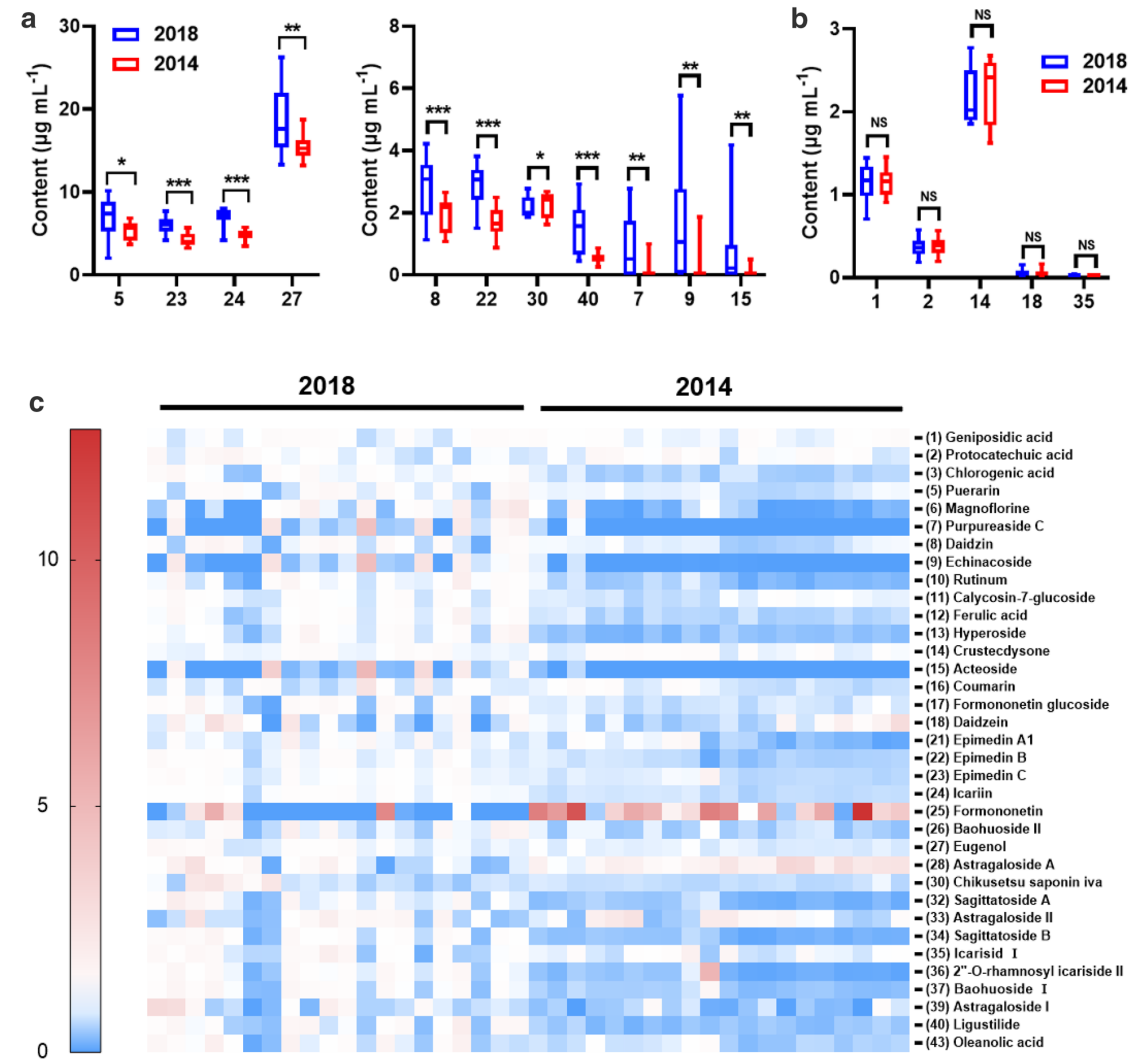
The content comparisons of the highlighted analytes (**a**) and the compounds without difference (**b**). Heatmap (**c**) visualizing the differences in the contents of the investigated analytes across the samples. The compound number in the *S*-plot represent in the same manner as in Table 1

In order to further visualize the difference between samples, OPLS-DA, a supervised multivariate data analysis, was further constructed to characterize the differences between groups on the basis of the levels of 35 analytes determined. As illustrated in Fig. 5a, the spirit samples produced at 2014 and 2018 were unambiguously segregated into two tight clusters (R^2^X = 0.826, R^2^Y  = 0.96, Q^2^ = 0.887), suggesting that the great alteration in the investigated compounds were presented. The cumulative values of R^2^X, R^2^Y, and Q^2^ were close to optimal value of 1.0, indicating the established models with excellent predictive capability and fitness [16]. To identify the differentiated components that contribute most to the group separation, the differentiated compounds were selected by *S*-plot derived from the constructed OPLS-DA. Eleven compounds with variable importance in the projection (VIP) of more than 1 (VIP > 1) and *p* value of less than 0.05 were highlighted in the *S*-plot (Fig. 5b). The group separation of the samples with the different production years could also be clearly classified in OPLS-DA score plot according to the contents of these 11 highlighted components (Fig. 5c). Compared to the spirit produced in 2018, the contents of the highlighted compounds, puerarin (**5**), purpureaside C (**7**), daidzin (**8**), echinacoside (**9**), acteoside (**15**), epimedin B (**22**), epimedin C (**23**), icariin (**24**), eugenol (**27**), chikusetsusaponin iva (**30**) and Z-ligustilide (**40**), significantly decreased in the samples produced in year 2014 (Fig. 6a). Geniposidic acid (**1**), protocatechuic acid (**2**), crustecdysone (**14**), daidzein (**18**) and icariside I (**35**) around the origin of *S*-plot were also highlighted as the stable compounds, which shown the comparable contents with less variation between samples from two production years (Fig. 6b). Additionally, a heatmap according to the relative contents of 35 detedcted analytes was constructed to display the changes in the analyte levels between groups (Fig. 6c).

## Conclusions

In this study, a rapid and sensitive UHPLC-QQQ-MS with MRM mode was developed to simultaneously quantify 43 bioactive components in Chinese herbal spirits. Quantitative results showed that 11 components, i.e.., puerarin (**5**), purpureaside C (**7**), daidzin (**8**), echinacoside (**9**), acteoside (**15**), epimedin B (**22**), epimedin C (**23**), icariin (**24**), eugenol (**27**), chikusetsusaponin iva (**30**) and Z-ligustilide (**40**), significantly decreased along with the increasing years of storage, while 5 compounds, i.e.., geniposidic acid (**1**), protocatechuic acid (**2**), crustecdysone (**14**), daidzein (**18**) and icariside I (**35**), were basically stable in all samples across the years. The established method allowing to simultaneously determined 43 components with wide structural diversity and trace amounts will facilitate the quality control research of Chinese herbal spirits.

## Supplementary Information

**Additional file 1: Figure S1.** Chemical structures of 43 investigated analytes.

## Data Availability

The data in this study are available from the corresponding author upon request.
