# Supplementary material for: Simultaneous quantification of bioactive components in Chinese herbal spirits by ultra-high performance liquid chromatography coupled to triple-quadrupole mass spectrometry (UHPLC–QQQ–MS/MS)
Source: Chin Med. 2021 Mar 12;16:26. doi: 10.1186/s13020-021-00435-0 (PMC7953818; doi:10.1186/s13020-021-00435-0)

**Additional file 1**

**Simultaneous Quantification of Bioactive Components in Chinese Herbal Spirits by Ultra-Performance Liquid Chromatography Coupled to Triple-Quadrupole Mass Spectrometry (UPLC–QQQ–MS/MS)**

Yan Hu ^a,b,1^, Zhe Wang ^c,1^, Fangbo Xia ^a^, Wen Yang^c^, Yuan-Cai Liu^c^, Jian-Bo Wan ^a,*^

^a^ State Key Laboratory of Quality Research in Chinese Medicine, Institute of Chinese Medical Sciences, University of Macau, Taipa, Macao SAR, China

^b^ State Key Laboratory Breeding Base of Systematic Research, Development and Utilization of Chinese Medicine Resources, School of Pharmacy, Chengdu University of Traditional Chinese Medicine, Chengdu, 611137

^c^ Hubei Provincial Key Lab for Quality and Safety of Traditional Chinese Medicine Health Food，Jing Brand Co.,Ltd. Hubei, China

^1^These authors contributed equally to this work

***Correspondences:**

**Dr. Jian-Bo Wan,**

E-mail: jbwan@um.edu.mo

Room 6034, Building N22, Institute of Chinese Medical Sciences,

University of Macau,

Avenida da Universidade, Taipa, Macao SAR, China

**Additional file 1: Figures**

**Fig. S1** Chemical structure of 43 investigated analytes.


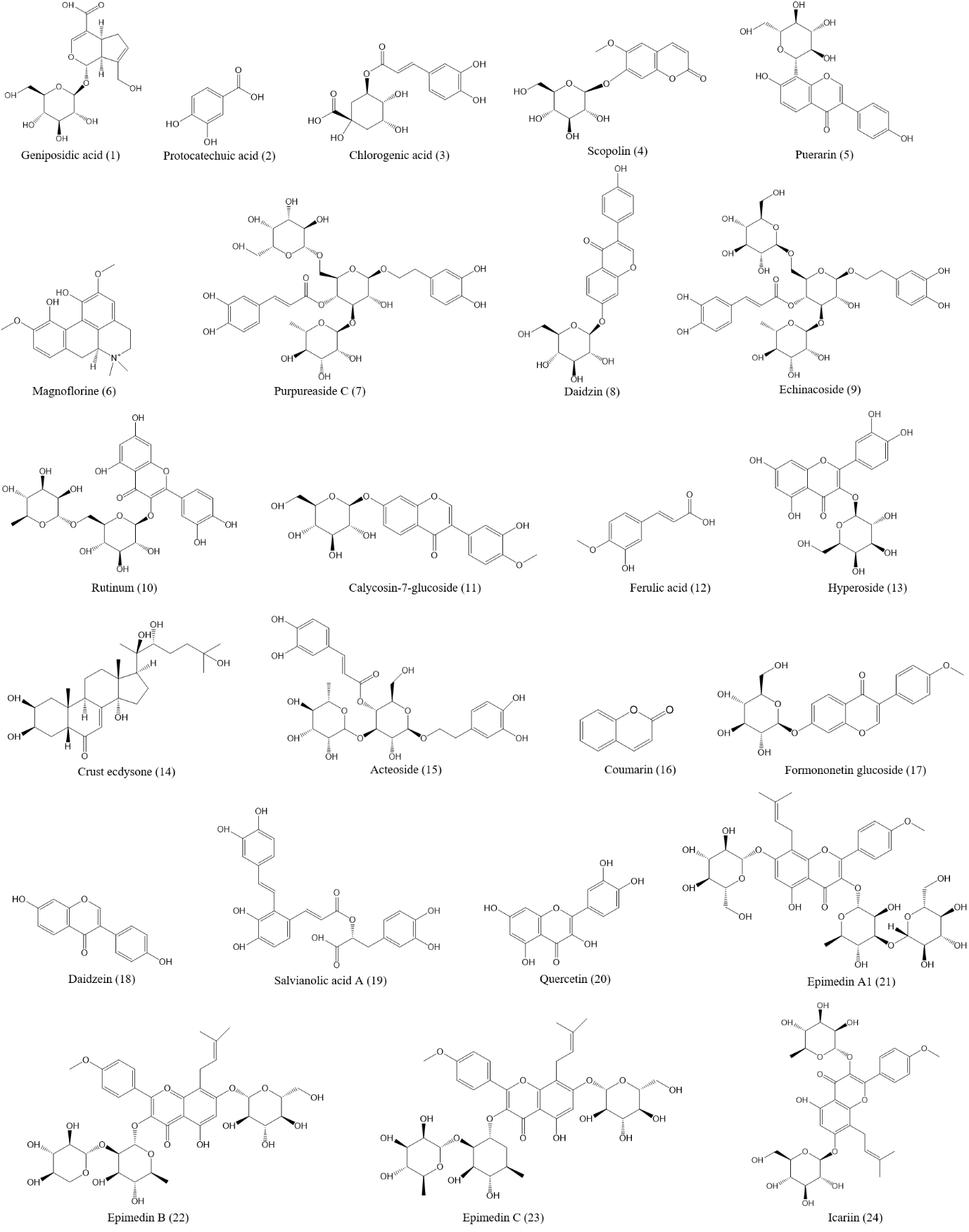


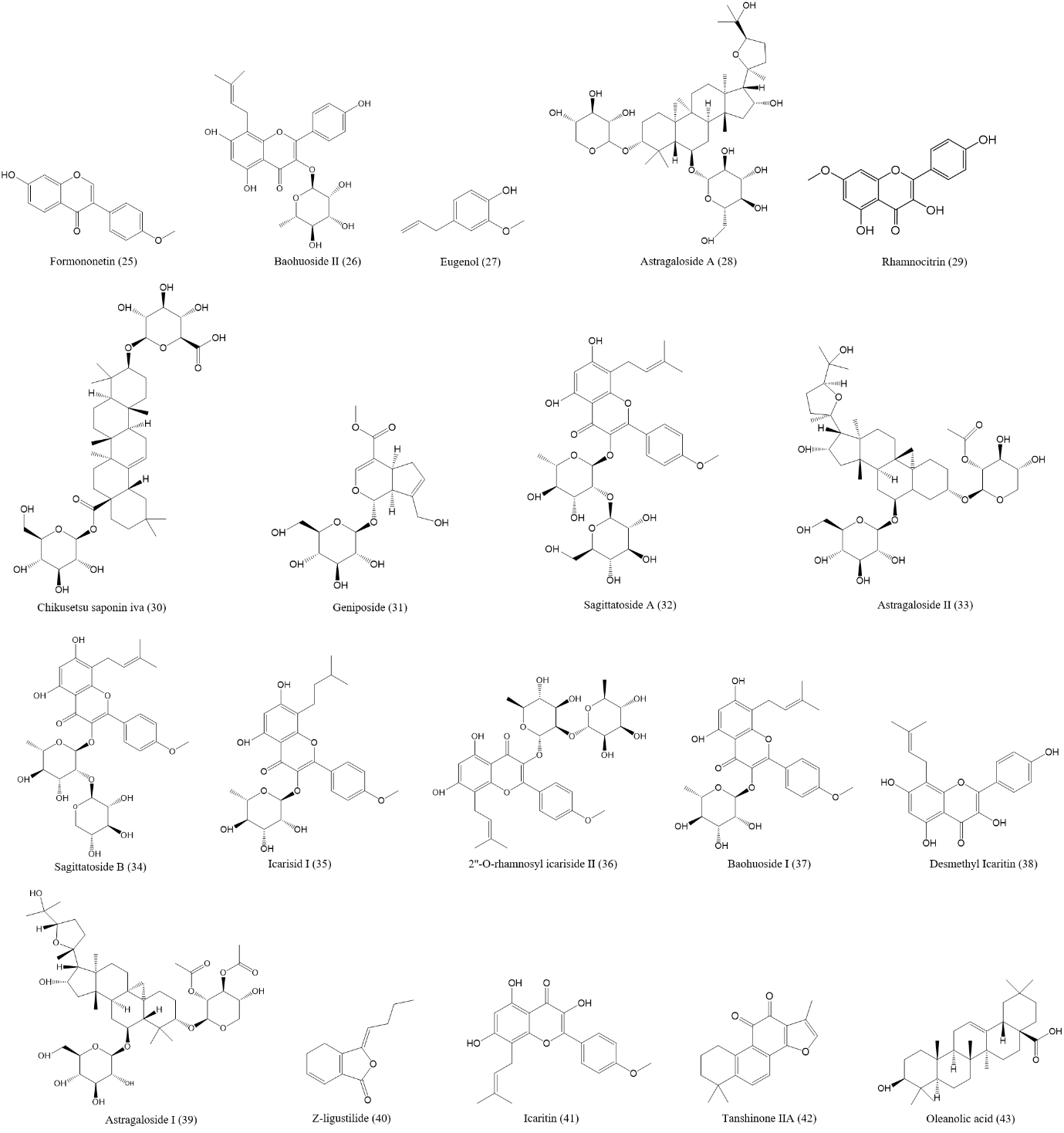

Supplement: Supplementary file 1 — Additional file 1: Figure S1. Chemical structures of 43 investigated analytes. [file 13020_2021_435_MOESM1_ESM.docx]
